# Efficacy and outcomes of surgical staples as an alternative to conventional skin closure in canine mastectomy

**DOI:** 10.1371/journal.pone.0332598

**Published:** 2025-09-19

**Authors:** Fábia Kariny Mendes Moreno, Gabriel Menezes Rodrigues, Victor Otero Martinez, Cinthia Oliveira de Araujo Barreto, Laís Pereira Silva, Thanielle Novaes Fontes, Vivian Fernanda Barbosa, João Moreira da Costa-Neto, Alessandra Estrela-Lima

**Affiliations:** 1 Research Center on Mammary Oncology (NPqOM), Federal University of Bahia, Salvador, Brazil; 2 Department of Veterinary Anatomy, Pathology and Clinics, School of Veterinary Medicine and Zootechny, Federal University of Bahia (UFBA), Salvador, Bahia, Brazil; 3 Postgraduate Program in Pharmacy - Federal University of Bahia (UFBA), Salvador, Bahia, Brazil; Ross University School of Veterinary Medicine, SAINT KITTS AND NEVIS

## Abstract

Surgical wound closure is critical in oncologic surgeries and directly influences healing, postoperative complications, and recovery time. In canine mastectomies, conventional skin closure with nylon sutures remains the standard technique; however, surgical staples have emerged as a potential alternative that can potentially optimize surgical efficiency and outcomes. This study evaluated the efficacy of surgical staples as an alternative to routine standard suture (nylon) in female dogs undergoing mastectomies. Fifty female dogs were divided into two groups: one was treated with nylon suture (NS group, n = 25) and the other with surgical staples (SS group, n = 25). Surgical time, local wound temperature, and wound healing characteristics were evaluated. Thermographic analysis of the surgical scar was performed on a subsample. Logistic regression and multivariate linear analysis were used to compare differences between the techniques, and an independent samples *t* test was applied to compare surgical and suture times. No significant differences were found between the groups regarding age, weight, incision size, body size, and reproductive status. Total surgical time was significantly shorter in the SS group (*p* = 0.011). The closure time was 9.8 times shorter in the SS group versus the NS group (p < 0.001). The average wound temperature was higher in the NS group (*p* < 0.001). The SS group showed fewer occurrences of wound alterations (*p* = 0.029) and a lower frequency of focal inflammatory exudate (*p* = 0.013). The thermographic analysis showed more white pixels and higher temperatures in the surgical wound in the NS group versus the SS group (*p* = 0.032 and *p* < 0.001, respectively). Surgical staples were found to be safe and effective for wound closure in mastectomies in female dogs, offering a viable alternative to traditional nylon suture techniques. Although the cost of staples is slightly higher, the benefits of shorter surgical time and reduced complications can make this technique a favorable choice, especially for oncologic patients with additional health concerns or surgical time restrictions.

## Introduction

Mammary neoplasms demonstrate a high prevalence in female dogs, accounting for 50–70% of all canine neoplasms; surgery is a key option for localized disease control, except in cases of inflammatory carcinoma, where surgery is not recommended [1–3]. The main surgical techniques include lumpectomy, regional mastectomy, and unilateral or bilateral mastectomies, with a goal to completely remove tumor masses with clear margins, thus reducing the recurrence risk [4,5]. 

Wound closure is essential to ensure surgical success and promote healing, as it stabilizes wound edges until tissue tensile strength is restored. Controlling tension and reducing dead space are critical challenges in mastectomy closures, especially in the inguinal region, where dehiscence risk is highest [6,7].

Wound closure success depends on the selection of appropriate materials with sufficient tensile strength, low tissue reactivity, and handling ease [8,9]. Nonabsorbable monofilament sutures, such as nylon, are traditionally used for mastectomy closure due to their low reactivity and wide availability in routine clinical practice. However, alternatives are continuously being explored [10]. Surgical staples stand out among these alternatives, as they were developed to save time and exhibit ideal characteristics for closure, including high strength and minimal tissue reactivity [11].

A meta-analysis of 13,661 cases indicated that using surgical staples reduces operating time across various surgeries. However, there is no clear evidence of superiority regarding surgical site infection, postoperative complications, or hospitalization duration [12]. Advances in veterinary medicine have led to the pursuit of techniques that reduce surgical time and enhance postoperative comfort; thus, this study was conducted to assess the efficacy of surgical staples in mastectomy closure in comparison with nylon sutures in female dogs.

## Materials and methods

### Ethical aspects and inclusion criteria

This research project was approved by the Animal Experimentation Ethics Committee of the School of Veterinary Medicine and Animal Science at the Federal University of Bahia (protocol no. 23/2021). All procedures were conducted in accordance with the Brazilian College of Animal Experimentation (COBEA) and Animal Research: Reporting of In Vivo Experiments (ARRIVE) guidelines for reporting animal research. All animals were domiciled and returned to their homes 24 hours after surgery and full recovery. The dogs’ owners were informed about the details of the research project through a signed Informed Consent Form.

All surgeries were therapeutic (indicated for the treatment of mammary tumors), and no animal was euthanized as part of this study. Pain was managed with multimodal analgesia, including opioids and NSAIDs, and the animals were monitored for signs of distress. No experiments were performed to induce disease or undertake additional invasive procedures beyond the therapeutic intervention.

Female dogs with mammary tumors requiring total unilateral mastectomy were included in this study, regardless of age or weight, and divided into two matched groups: the nylon suture group (NS), comprising dogs undergoing skin closure with 3-0 nylon (Procare®, Rio de Janeiro, Brazil) using interrupted simple sutures (n = 25) and the surgical staple group (SS), comprising dogs undergoing skin closure with surgical staples (Guanchuang®, China) (n = 25).

Animals were excluded if they presented: (1) clinical or laboratory evidence of systemic infections or severe organ dysfunction (hepatic, renal, or cardiac failure); (2) inflammatory or ulcerative skin lesions at the surgical site; or (3) signs of advanced cachexia or poor general condition. These criteria aimed to minimize factors interfering with wound healing and postoperative evaluation.

### Preoperative evaluation and anesthesia

All dogs underwent a general clinical examination, including the evaluation of physiological parameters and a specific examination of the mammary chain, which involved palpation, an assessment of axillary and inguinal lymph nodes, and tumor measurement. As part of the preoperative evaluation, imaging exams (such as thoracic radiographs and abdominal ultrasound), hematological and biochemical tests, and cardiac assessments (electrocardiogram and echocardiogram) were conducted to determine the animal’s surgical and anesthetic risk and to detect possible metastases.

Clinical staging followed the guidelines from the consensus on mammary neoplasms [1], which consider tumor size (T), sentinel lymph node involvement (N), and the presence of distant metastases (M).

In preoperative preparation, the dogs underwent 8 hours of fasting from food and 1 hour from water. The anesthetic protocol was adjusted according to each patient’s characteristics, classified as ASA II per their oncological condition and, in most cases, advanced age with controlled systemic disease. Initially, the dogs were medicated intramuscularly (IM) with 0.03 mg/kg of acepromazine (Acepram® 0.2%, Vetnil, São Paulo, Brazil) and 0.3 mg/kg of morphine sulfate (Hipolabor, Minas Gerais, Brazil). After venous access and hair removal from the abdominal region, induction anesthesia was performed with propofol (Propovan®, Cristália, São Paulo, Brazil) at a dose of 2 mg/kg intravenously, followed by maintenance with isoflurane (Isoforine®, Cristália, São Paulo, Brazil) diluted in medical oxygen at 100%. Subsequently, locoregional blockade of the mammary chain was performed using the tumescent technique [13]. The solution used comprised 40 ml of lidocaine without a vasoconstrictor and one ampoule of adrenaline (Adren®, Hipolabor, Minas Gerais, Brazil) at a concentration of 1 mg/ml in 460 ml of saline solution; 15 ml/kg was injected subglandularly along the entire mammary chain to be removed.

### Surgical procedure

All procedures were performed by the same surgical team, and the operative time was recorded from the start of the incision until the completion of suturing or stapling. The skin closure time was recorded separately. For unilateral total mastectomy (UTM), incisions were made parallel to the affected mammary chain, with surgical margins extending approximately 2–3 cm beyond the palpable tumor edges. The subcutaneous tissue was dissected from the thoracic mammary glands to the abdominal and inguinal glands. The caudal superficial epigastric vein was ligated with poliglecaprone 25-PGCL (Bioline®, São Paulo, Brazil) and removed with the mammary chain and inguinal lymph node. The surgical site was inspected for hemostasis and the potential need for additional tissue removal. The incision was closed in two layers: a continuous intradermal suture using PGCL and a skin closure with either interrupted nylon sutures (NS group) or surgical staples (SS group). In the NS group, interrupted simple sutures were placed with nylon 3-0 (Procare®, Rio de Janeiro, Brazil). In the SS group, the wound edges were held with Adson Brown forceps, and a stapler (GCPFA/B/C-35, Guanchuang®, China) was used to close the skin incision by approximating the wound edges with staples. The removed mammary chain was placed in formalin and sent for histopathological evaluation.

### Postoperative care

The patients were hospitalized for 24 hours. Postoperative care included analgesia with subcutaneous methadone every 8 hours (Metadon® 10 mg/ml, Cristália, São Paulo, Brazil) at 0.5 mg/kg, intramuscular antibiotic amoxicillin with potassium clavulanate every 24 hours (Agemoxi®, Agener União, São Paulo, Brazil) at 20 mg/kg, intramuscular anti-inflammatory meloxicam every 24 hours (Maxicam® 0.2%, Ourofino, São Paulo, Brazil) at 0.1 mg/kg, and oral gastric protectant omeprazole every 12 hours (Petprazol®, Vetnil, São Paulo, Brazil) at 1 mg/kg. The same medications were prescribed for home administration apart from methadone, which was replaced with oral tramadol hydrochloride (Cronidor® Agener União, São Paulo, Brazil) at 3.5 mg/kg.

The surgical wound was cleaned once daily with 0.5% chlorhexidine digluconate solution (Merthiolat®, Cosmed Indústria de Cosméticos e Medicamentos S.A., São Paulo). Compressive bandages were placed for 24 hours, and the dogs wore surgical clothing and Elizabethan collars for 15 days after the surgery, at which point the sutures or staples were removed.

### Clinical evaluations

**Fig 1:**
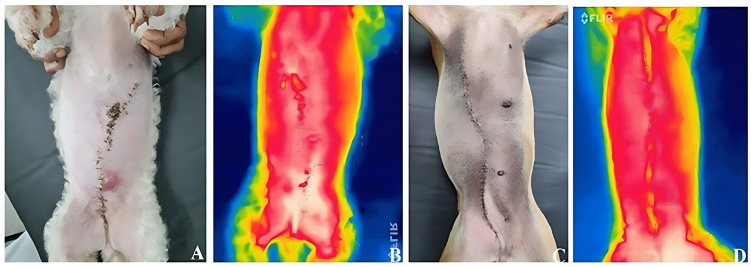
Photographic and thermographic images of surgical scars in female dogs on postoperative day 7. (A, B) Dog with nylon sutures: (A) photographic image and (B) corresponding thermographic image; (C, D) Dog with staples: (C) photographic image and (D) corresponding thermographic image.

On postoperative day 7, all surgical wounds were evaluated per an evaluation form for clinical signs of inflammation or complications. The recorded variables included erythema, edema (increased volume), exudate, dehiscence, and crust formation. These findings were grouped under the variable “changes in the surgical wound” and classified according to their severity as mild (e.g., discreet redness or swelling without exudate), moderate (e.g., evident inflammation and limited serous exudate), or severe (e.g., marked inflammation, purulent discharge, or dehiscence). The skin temperature around the incision was measured with an infrared digital thermometer and compared with the temperature of the skin 2 cm from the incision (Fig 1). Patients with high-grade tumors or metastasis to regional lymph nodes were referred for oncological follow-up and chemotherapy.

Additionally, thermographic images of the surgical wounds were obtained using a FLIR® E6 infrared camera (FLIR Systems, USA), with a resolution of 160 × 120 pixels and thermal sensitivity of <0.06°C. Images were captured at a standardized distance of 50 cm from the incision site, perpendicular to the body surface, with animals in the standing position. Thermograms were acquired on postoperative day 7 and analyzed using FLIR Tools® software to quantify pixel distribution by color range (white, red, yellow), reflecting temperature variation.

### Cost evaluation

A cost analysis of the materials was performed to assess the financial impact associated with the use of surgical staples. A surgical stapler with 35 staples costs R$35.00 (approximately USD 6.50) on average, whereas a nylon suture package costs approximately R$2.00 (approximately USD 0.40). The budget evaluation considered the size of the animals and the average number of units used per procedure. Additionally, costs were compared between the suture techniques (nylon suture and surgical staple) to determine the financial difference between the approaches. The analysis also considered the need for material replacement in cases of technical failure, impacts on hospitalization time, and the occurrence of wound complications necessitating additional postoperative interventions, such as topical treatments or extended antibiotic use.

### Data analysis

The data were tabulated, and frequency, central tendency, and dispersion measures were analyzed. Parametric or nonparametric tests were selected according to the Kolmogorov–Smirnov normality test. Fisher’s exact test was used to compare the techniques employed, while an independent samples *t* test was applied to compare the mean surgical times and temperatures between the groups. A paired *t* test was used to compare the time and temperature results within the same animal (chain 1 *vs.* chain 2). The Mann–Whitney U test was applied to compare the percentage of pixels in thermographic images. For the postoperative data analyses, binary logistic regression was used to compare the groups in terms of clinical evaluation. The odds ratio (OR) was calculated from the exponentiation of the beta coefficient obtained in the multivariate model, adjusted for histopathological covariates, weight, age, reproductive status, size, surgical time, skin closure, and incision size. Linear regression analysis was used to compare the times and local temperatures between the groups, and the adjusted R² was calculated to verify the percentage of variation explained by the linear model. Using two-tailed analysis, the significance level adopted was *p* < 0.05, with a 95% confidence interval. Statistical analyses were performed using SPSS® 26.0 for Windows and GraphPad Prism 8.0.2.

## Results

A total of 50 surgical procedures were completed without any complications. None of the animals required reintervention to replace the skin closure materials. Furthermore, no suture or staple dehiscence cases were observed in any of the animals included in this study.

No significant differences were observed between the two groups regarding age (NS: 10.1 ± 0.4 years; SS: 9.9 ± 0.4 years), weight (NS: 9.3 ± 1.7 kg; SS: 9.9 ± 1.3 kilograms), incision size (NS: 31.4 ± 1.5 cm; SS: 32.9 ± 1.4 cm), reproductive status, and tumor histological type, indicating group homogeneity in terms of main characteristics.

The total surgical time showed significant differences between the groups *(p* = 0.001). Furthermore, there was a substantial difference in skin closure time, with the NS group showing an average closure time 9.8 times greater than that in the SS group *(p* < 0.001). The average temperature of the surgical wound was higher in the NS group (mean = 37.89ºC) compared with that in the SS group (mean = 37.1ºC), and the difference was significant (*p* < 0.001) (Table 1).

**Table 1:** Main characteristics of the surgical procedures and the female dogs undergoing skin closure after mastectomy according to the group.

Group	Nylon Suture (n = 25)	Surgical Staple (n = 25)	p-value
Variable	Mean (± SD)	Mean (± SD)	
**Age (years)**	10,1 (±0,4)	9,9 (±0,4)	0,790
Minimum	7	5	
Maximum	14	14	
**Weight (Kg)**	9,3 (±1,7)	9,9 (±1,3)	0,789
Minimum	1,4	3,3	
Maximum	37,1	31,0	
**Surgical time (min)**	67,1 (±3,8)	48,8 (±3,1)	0,001*
Minimum	37,0	31,0	
Maximum	95,0	81,0	
**Skin closure time (min)**	11,8 (±0,7)	1,2 (±0,05)	<0,001*
Minimum	7,8	0,9	
Maximum	22,0	2,0	
**Incision size (cm)**	31,4 (±1,5)	32,9 (±1,4)	0,530
Minimum	22	22	
Maximum	48	48	
**Temperature at site (ºC)**	37,9 (±0,11)	37,1 (±0,12)	<0,001*
Minimum	36,7	36,2	
Maximum	38,7	38,9	
**Variable**	**n (%)**	**n (%)**	
**Histopathological**			
Benign Tumors	2 (8,0)	4 (16,0)	0,531
Malignant Tumors I	13 (52,0)	15 (60,0)	
Malignant Tumors II	5 (20,0)	2 (8,0)	
Malignant Tumors III	5 (20,0)	4 (16,0)	
**Size**			
Small (0,1–10 kg)	21 (84,0)	15 (60,0)	0,233
Medium (10,1–25 kg)	2 (8,0)	9 (36,0)	
Large (> 25,1 kg)	2 (8,0)	1 (4,0)	
**Neutered**			
Yes	10 (40,0)	11 (44,0)	0,774
No	15 (60,0)	14 (56,0)	

Statistical tests: Student’s T-test and Fisher’s exact test. * Statistically significant different.

Surgical wound assessment was conducted on postoperative day 7 to identify early healing alterations. A logistic regression analysis was used to evaluate the association between closure type (staples or nylon sutures) and the presence of postoperative wound alterations (Table 2). The histopathological outcome variables, weight, age, reproductive status, size, surgical time, skin closure time, and incision size, were considered to adjust the multivariate model. No animal showed immediate complications or dehiscence in the surgical wound. Although no dehiscence occurred, 12 female dogs returned with the loss of 1–4 surgical staples.

**Table 2:** Comparison between the groups according to the evaluation of the surgical wound in female dogs undergoing mastectomy after seven days (first post-surgical consultation) based on multivariate models by logistic regression.

Group	Nylon Suture (n = 25)	Surgical Staple (n = 24)	OR (IC 95%)	p-value
Changes in the surgical wound**				
No	0	7 (29,2)	Ref.	0,029*
Yes	25 (100)	17 (70,8)	11,3 (1,3–98,9)	
**Occurrence of dehiscence**				
No	25 (100)	25 (100)	**	**
Yes	0	0	**	
**Degree of severity of changes**				
Absence/Mild	19 (76,0)	17 (70,8)	Ref.	0,682
Moderate/Severe	6 (24,0)	7 (29,2)	9,9 (1,1–87,9)	
**Hyperemia**				
No	2 (8,0)	15 (62,5)	Ref.	0,001*
Yes	23 (92,0)	9 (37,5)	19,2 (3,6–101,3)	
**Crusts**				
No	0	10 (41,7)	Ref.	0,010*
Yes	25 (100)	14 (58,3)	17,1 (2,0–148,4)	
**Ulceration**				
No	24 (96,0)	24 (100)	Ref.	0,338
Yes	1 (4,0)	0	3,1 (0,3–32,5)	
**Increased volume**				
No	19 (76,0)	24 (100,0)	Ref.	0,078
Yes	6 (24,0)	0	7,3 (0,8–65,7)	
**Bleeding**				
No	18 (72,0)	23 (95,8)	Ref.	0,092
Yes	7 (28,0)	1 (4,2)	4,3 (0,8–23,2)	
**Seroma**				
No	20 (80,0)	23 (95,8)	Ref.	0,124
Yes	5 (20,0)	1 (4,2)	5,7 (0,6–53,4)	
**Inflammatory exudate**				
No	15 (60,0)	24 (100)	Ref.	0,013*
Yes	10 (40,0)	0	15,3 (1,8–132,4)	

* Statistically significant association. ** Changes in the surgical wound include erythema, edema (recorded as increased volume), exudate, dehiscence, and crust formation. Statistical tests: Multivariate logistic regression. Covariates: Histopathological, weight, age, reproductive status, size, surgical time, skin closure time, and incision size. Ref. – Reference group.

**Fig 2:**
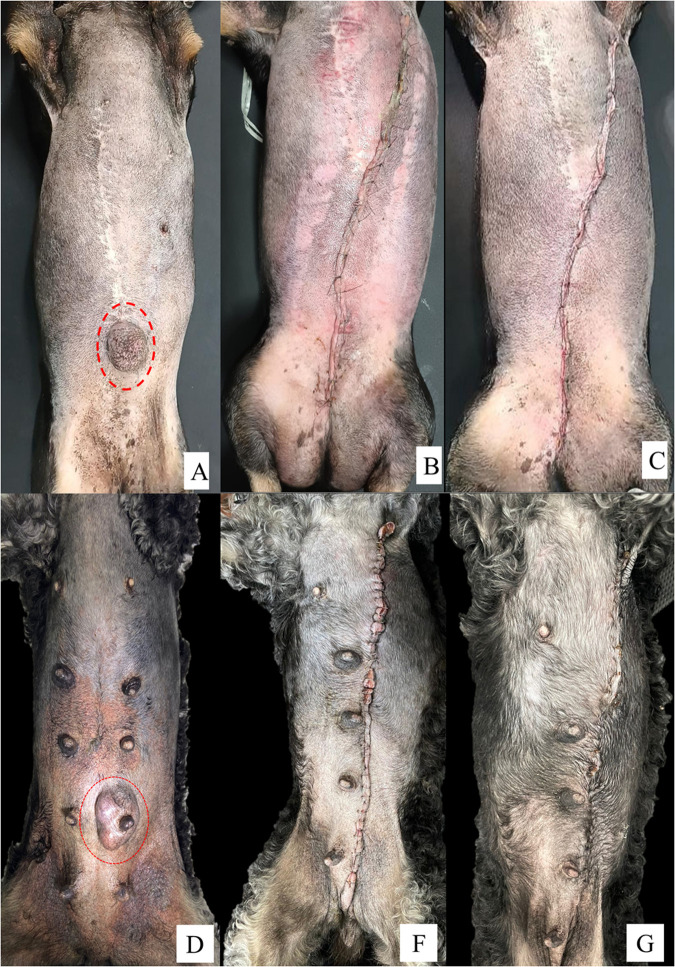
Clinical evolution of surgical wounds in female dogs undergoing mastectomy with nylon sutures and surgical staples. (A–C) Patient undergoing mastectomy with nylon sutures: (A) preoperative aspect of the mammary chain; mammary tumor highlighted by the red circle; (B) aspect of the immediate postoperative wound; (C) aspect of the wound 15 days after surgery. (D–F) Patient undergoing mastectomy with surgical staples: (D) preoperative aspect of the mammary chain; mammary tumor highlighted by the red circle; (E) aspect of the immediate postoperative wound; (F) aspect of the wound 15 days after surgery.

The NS group showed an 11.3 times higher OR (95% CI, 1.3–98.9) for developing wound alterations (*p* = 0.029). In this study, “wound alteration” was defined as the occurrence of one or more of the following signs during clinical evaluation: hyperemia, crust formation, ulceration, increased volume, bleeding, seroma, or inflammatory exudate. Compared with the NS group, the SS group exhibited a significantly lower incidence of hyperemia (*p* = 0.001) and crust formation (*p* = 0.010). Furthermore, the multivariate analysis indicated that female dogs in the NS group were 15.3 times more likely to develop inflammatory exudate than were those in the SS group (OR = 15.3; 95% CI, 1.8–132.4; *p* = 0.013). The clinical progression of surgical wounds 15 days post-mastectomy for both closure techniques is illustrated in Fig 2.

The costs related to the use of materials were calculated according to the size of the dogs. Only one stapler with 35 staples, or about five packages of nylon suture, was used for small animals (≤10 kg). For medium-sized dogs (10–25 kg), approximately 50 staples (1 stapler with 35 staples and one with 15 staples) or 10 packages of nylon sutures were required. Large dogs (>25 kg) necessitated two staplers with 35 staples or 15 packages of nylon sutures, on average. In the skin closure material cost comparison, staples were found to increased expenses by 233.3% for large animals, 262.5% for medium-sized animals, and 350% for small animals.

The multivariate model results showed that the NS group had significantly longer surgical duration than the SS group (*p* = 0.001). Additionally, the type of material used in skin closure explained 72.3% of the variation in surgical time. Skin closure time was longer in the NS group, regardless of the dogs’ tumor type, weight, age, size, or reproductive status (*p* < 0.001). The linear regression analysis confirmed significant differences between the groups (*p* < 0.001) regarding local temperature. The NS and SS group mean temperatures were 37.9 (±0.11) and 37.1 (±0.12), respectively. The multivariate analysis showed that the variables analyzed explained 48.6% of the variation in local temperature (Table 3). Histopathological variables, weight, age, reproductive status, and size were used to adjust the model.

**Table 3:** Comparison of surgical times, skin closure time, and temperature at the surgical site in female dogs undergoing mastectomy, based on multivariate linear regression models.

Group	Nylon Suture (n = 25)	Surgical Staple (n = 25)	R²	p-value
Variable	Mean (± SD)	Mean (± SD)		
**Surgical time (min)**	67,1 (±3,8)	48,8 (±3,1)	0,723	0,001*
Minimum	37,0	31,0		
Maximum	95,0	81,0		
**Skin closure time (min)**	11,8 (±0,7)	1,2 (±0,05)	0,841	<0,001*
Minimum	7,8	0,9		
Maximum	22,0	2,0		
Temperature at site (ºC)**	37,9 (±0,11)	37,1 (±0,12)	0,486	<0,001*
Minimum	36,7	36,2		
Maximum	38,7	38,9		

* Statistically significant association. Statistical tests: Linear regression. Covariates: Histopathological findings, weight, age, reproductive status, and size.

**Temperature data refer to measurements taken on the seventh postoperative day.

Among the dogs participating in the study, six had tumors in both mammary chains. The second chain was removed 60 days after the first. A paired analysis was performed using a different type of material for each mammary chain to assess whether there were significant differences in surgical time, skin closure time, and local temperature in the same animals. The differences observed among the variables remained significant except for total surgical time, which did not differ according to the type of material used for skin closure (Table 4).

**Table 4:** Paired comparison of surgical time, skin closure time, and local temperature in six female dogs undergoing bilateral mastectomy.

Group			Surgical time (min)	Skin closure time (min)	Local temperature (ºC)**	
			Média (± DP)	Média (± DP)	Média (± DP)	
Surgical Staple (n = 6)			51.0 (± 19.4)	1.08 (± 0.11)	37.3 (± 0.26)	
Nylon Suture (n = 6)			63.7 (± 22.0)	11.3 (± 2.04)	38.2 (± 0.26)	
**p – value**			0.315	< 0.001*	< 0.001*	
**Patient Data**						
	Chain 1 – Staple	Chain 2 – Nylon suture	Chain 1 – Staple	Chain 2 – Nylon suture	Chain 1 – Staple	Chain 2 – Nylon suture
Patient	Surgical time (min)		Skin closure time (min)		Local temperature (ºC)**	
**1**	32	41	1.17	10.63	37.2	38.2
**2**	80	50	0.98	8.24	37.5	38.3
**3**	70	41	0.92	12.00	37.1	38.6
**4**	38	78	1.20	14.50	36.9	37.8
**5**	40	85	1.12	10.77	37.3	38.1
**6**	46	87	1.08	11.45	37.6	38.3

* Statistically significant association. Statistical tests: Paired t-test.

**Temperature data refer to measurements taken on the seventh postoperative day.

After defining the areas of interest for each tumor according to its spatial extent, the quantity of white (indicating higher temperature), red, and yellow (indicating lower temperature) pixels from each thermogram was individually extracted, and the percentage of coverage was evaluated.

The animals in the NS group had a white pixel (PB) coverage area ranging from 9% to 53%, with a median of 37%. The median for red pixels (PV) was 42%, with coverage ranging from 31% to 52%, and the median for yellow pixels (PA) was 7%, with coverage ranging from 5% to 35%. The SS group had a median of 4.9% PB, ranging from 0% to 19.5%, a median of 51.0% PV with coverage ranging from 11.9% to 65.4%, and 5.9% PA, with coverage ranging from 0% to 18%.

**Fig 3:**
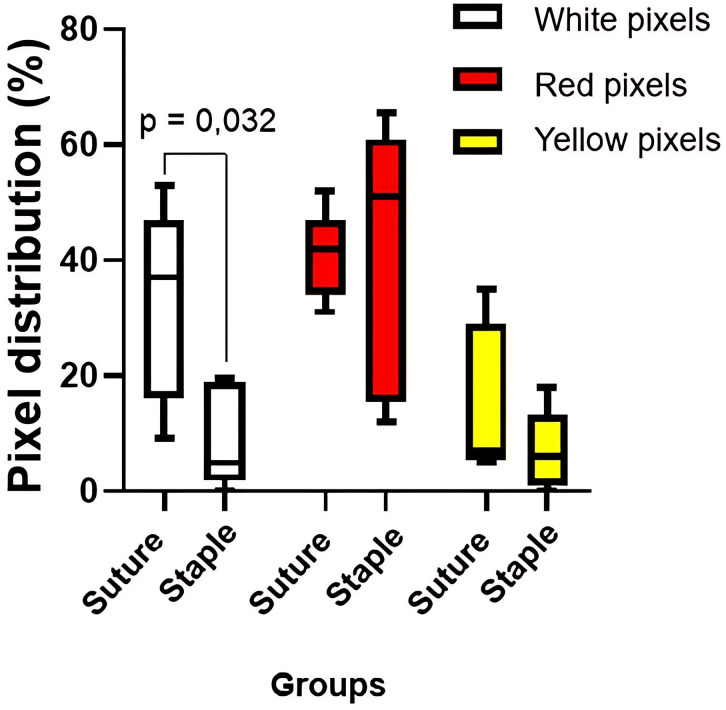
Box plot of the distribution of white, red, and yellow pixels in the experimental groups. In the thermographic images, white pixels represent areas of higher temperature (increased local inflammation) along the surgical wound, red pixels represent intermediate temperatures, and yellow pixels indicate areas with lower temperature.

There was a significant difference in PB coverage between the NS and SS groups (*p* = 0.032), indicating a direct relationship between the increase in PB coverage and the type of material used for skin closure. No significant differences were found between the groups in terms of PV and PA coverage (Fig 3). A thermal disparity between the surgical scars of the NS and SS groups was observed in the qualitative thermogram assessment (Fig 1).

## Discussion

The use of surgical staples for mastectomy incision skin closure in female dogs was evaluated and compared with the use of nylon sutures in a simple interrupted pattern. Despite their relatively higher cost, surgical staples may present a viable alternative for this procedure, particularly in large-breed dogs. The two groups evaluated in the study demonstrated no significant differences in key clinicopathological characteristics, indicating homogeneity in the variables, ruling out discrepancies that could have influenced the assessments.

The findings of this study support our initial hypothesis that surgical staples would provide improved surgical efficiency and better postoperative outcomes than conventional nylon sutures in canine mastectomies. The staples resulted in shorter dermal closure times, reduced wound temperature, and a lower frequency of postoperative alterations, reinforcing their potential as a viable alternative for skin closure in oncologic veterinary surgery.

The total surgical and skin closure durations in the SS group were significantly shorter than those in the NS group. Multivariate regression modeling and paired analysis further confirmed reduced skin closure time with staples. These findings are consistent with those reported in a systematic review and meta-analysis that demonstrated significantly reduced closure time with surgical staples versus sutures in orthopedic surgeries, despite no significant differences in infection rates or other secondary outcomes [14]. Female dogs undergoing mammary gland removal are often cancer patients, typically elderly and affected by comorbidities such as cardiac, renal, or hepatic disease, which makes shorter surgical time especially important for reducing anesthetic and postoperative risk [15,16]. The use of surgical staples for mastectomy skin closure would also benefit dogs with pulmonary metastasis. It is known that animals undergoing longer surgeries and extended exposure to anesthetics and analgesics have a higher rate of postoperative hypothermia and require prolonged immediate postoperative recovery [17,18].

The use of staples for skin closure may also reduce postoperative pain. Although this study did not evaluate postoperative pain, the shorter closure time and reduced tissue manipulation observed with staples may improve patient comfort. In human medicine, mastectomy is classified as a medium to high pain intensity surgery [19], and this classification is considered applicable to dogs due to the similarly invasive nature of the procedure, which involves extensive tissue manipulation and wide surgical margins. Skin closure in mastectomy requires more tissue manipulation, which is directly linked to postoperative pain intensity and wound healing success [5]. Thus, reducing the time spent on wound closure and minimizing tissue manipulation may result in reduced postoperative pain.

The surgical team observed qualitative variations in the nylon suture points during the procedures, including inconsistencies in tension, the spacing between stitches, and the visual thickness of the suture line due to overlapping or poorly adjusted knots. Although not quantitatively measured, these observations were systematically noted as part of the clinical evaluation process. These variations may interfere with tissue vascularization at the wound margin, potentially leading to complications such as necrosis [20,21]. In contrast, staples are manufactured following a standardized process, ensuring greater uniformity in their application. Notably, the number of suture points tends to be higher than the number of staples, resulting in greater tissue trauma due to increased perforations and the accumulation of suture material (threads and knots) within the tissue, which may elicit a more intense local inflammatory response. Additionally, the suture technique is more invasive and traumatic than staple use due to the need for forcep manipulation and needle perforation [22].

To our knowledge, only one published study has specifically evaluated the use of surgical staples for skin closure following mastectomy in dogs, which initially makes the comparison of results in this species challenging. However, in humans, a prospective study involving 60 patients undergoing mammoplasty or abdominoplasty evaluated the use of absorbable staples (Insorb®), concluding that this method reduced surgical time by seven times without compromising scar quality or patient comfort [23]. Consistent with our findings, a meta-analysis evaluating six studies concluded that stapled skin closure reduced surgical time compared with that required for intradermal sutures [11].

Significant differences in local postoperative tissue responses were observed between incisions closed with nylon sutures and those closed with surgical staples, including higher frequencies of scabs, erythema, and focal inflammatory exudate in the NS group. While 68% of wound changes were recorded in the SS group, 100% of the female dogs in the NS group exhibited some alteration, primarily erythema and scabs. A study in rats demonstrated that nylon sutures used in internal tissues induced a greater inflammatory response than poliglecaprone 25, likely due to the nonabsorbable nature of nylon [24]. Although cutaneous use was not directly evaluated, these findings suggest that the suture material may influence local tissue reactivity. In contrast, other human and veterinary medicine studies have indicated that staples are associated with minimal tissue reactivity and may promote more uniform healing with lower local inflammation [25].

The presence of inflammatory exudate, used in this study as an indicator of local inflammatory response rather than a definitive sign of infection, was less frequent in the SS group. Importantly, secretion was confined to localized areas and did not extend across the entire wound, with small volumes of exudate produced. In a comprehensive systematic review with meta-analysis [12], no evidence was found to suggest that staples reduce the likelihood of infection at the surgical site. Thus, given the subjective evaluation performed in this study, further trials with specific methodologies assessing surgical wound infection will be essential to determine the viability of staple use in reducing infections in clinical practice.

The present study indicates the safety of using staples in skin closure, and no wound dehiscence was observed, consistent with the findings of a previous study [25] evaluating staple use versus subcuticular sutures in knee replacement surgeries. However, the closure techniques differed, as our protocol included intradermal sutures in both groups. The staple failure rate was low in the aforementioned study, consistent with the findings of the present study, in which no cases of dehiscence were recorded. Furthermore, another study [26] compared surgical clips with interrupted nylon sutures in human orthopedic wound closure, with no significant difference in healing time. However, the type of closure was found to influence wound appearance, with staples producing a more regular and uniform scar.

Surgeons should aim for uniform pressure, the proper approximation of wound edges, symmetry, and consistent spacing between sutures throughout the wound [20,27]. Surgical staples can help facilitate more standardized wound closure, offering consistent depth and shape. However, even spacing still depends on proper technique during application. Staples approximate the wound edges without requiring knots or additional tension across the incision, and their design allows them to remain slightly elevated above the skin surface, potentially reducing direct pressure on the wound margins.

A key finding of this study was that temperature at the surgical wound site was lower in the SS group than that in the NS group, suggesting a reduced inflammatory response and potentially less postoperative pain with staples. The multivariate linear regression model adjusted for tumor type, weight, age, reproductive status, and wound size explained 48.6% of the variation in local temperature after 7 days, reinforcing the impact of the suture material on inflammation. Inflammation increases temperature due to vasodilation and elevated blood flow [28]; thus, this finding is consistent with the thermographic analysis, which showed a significantly higher frequency of white pixels, indicative of elevated temperature, in the NS group. These results suggest that staples may be a more favorable option for skin closure, as they are associated with a lower inflammatory response [29,30]. Additionally, the use of infrared thermography as a non-invasive tool was effective in detecting these differences, supporting its value in surgical wound assessment [31,32].

Despite the valuable insights gained, this study has some limitations. First, no formal power analysis was performed before determining the optimal sample size, which could impact the generalizability of the findings and the ability to detect more minor yet clinically significant differences between the groups. Although significance was observed for several key outcomes, future studies would benefit from a power calculation to ensure an adequate sample size for detecting specific effect sizes. Second, although the wound healing evaluations were standardized and performed by the same surgical team, the subjective nature of some clinical assessments (e.g., the visual scoring of hyperemia or scabs) could have introduced a degree of observer bias. Efforts were made to ensure homogeneity between the groups regarding various characteristics; however, the inherent variability in individual animal responses to surgery and healing processes remains a potential confounding factor. Another limitation is the absence of microbiological culture and sensitivity testing to confirm whether the observed inflammatory exudate was associated with infection. This testing could provide a more accurate assessment of postoperative complications. Finally, the study was conducted at a single institution, which might limit the external validity of the results. Future multicentric studies with larger sample sizes and blinded assessments would further strengthen the evidence regarding the efficacy and outcomes of surgical staples versus nylon sutures in canine mastectomies.

Using surgical staples represents a significant additional cost. This cost increase is more pronounced for small versus large-breed dogs. However, the material offers several advantages over nylon suture, such as reduced skin closure time and a lower frequency of postoperative alterations, which may help prevent additional costs related to extended care or further interventions. Additionally, staples may be used in cases where patients require reduced surgical time due to pulmonary metastasis or comorbidities. In conclusion, considering the observed benefits, the use of staples in canine mastectomy closures is expected to become more common. As more veterinary surgeons gain experience and confidence with the technique, this method could be increasingly adopted for selected procedures.

## Conclusions

Surgical staples offer a rapid, uniform, and effective solution for skin closure in canine mastectomies, particularly benefiting patients with mammary tumors with higher surgical risks due to age and comorbidities. The reduced surgical and closure times provided by staples may help shorten the anesthesia duration, which is especially valuable for patients with pulmonary metastasis or cardiovascular issues. Additionally, staples were associated with reduced local inflammation and fewer postoperative wound alterations. Although initially more costly, this technique may help prevent complications and reduce the need for follow-up care. As experience with this method increases, it may become a helpful option in selected oncologic surgeries requiring efficient and safe wound closure.

## Supporting information

S1 Table Clinical-pathological data and surgical wound characteristics of animals in the nylon sutures group (n = 25) and the surgical sutures group (n = 25). (XLSX)
